# Sedanolide Activates KEAP1–NRF2 Pathway and Ameliorates Hydrogen Peroxide-Induced Apoptotic Cell Death

**DOI:** 10.3390/ijms242216532

**Published:** 2023-11-20

**Authors:** Yosuke Tabei, Hiroko Abe, Shingo Suzuki, Nobuaki Takeda, Jun-ichiro Arai, Yoshihiro Nakajima

**Affiliations:** 1 Health and Medical Research Institute, National Institute of Advanced Industrial Science and Technology (AIST), 2217-14 Hayashi-cho, Takamatsu 761-0395, Kagawa, Japan; y-tabei@aist.go.jp (Y.T.); abe-abe@aist.go.jp (H.A.); suzuki.shingo@kagawa-u.ac.jp (S.S.); 2 Department of Anatomy and Neurobiology, Faculty of Medicine, Kagawa University, 1750-1 Ikenobe, Miki-cho 761-0793, Kagawa, Japan; 3 Technology and Innovation Center, Daikin Industries, Ltd., 1-1 Nishi-Hitotsuya, Settsu 566-8585, Osaka, Japan; nobuaki.takeda@daikin.co.jp (N.T.); junichirou.arai@daikin.co.jp (J.-i.A.)

**Keywords:** sedanolide, ARE, NRF2, oxidative stress, apoptosis

## Abstract

Sedanolide is a bioactive compound with anti-inflammatory and antitumor activities. Although it has been recently suggested that sedanolide activates the nuclear factor E2-related factor 2 (NRF2) pathway, there is little research on its effects on cellular resistance to oxidative stress. The objective of the present study was to investigate the function of sedanolide in suppressing hydrogen peroxide (H_2_O_2_)-induced oxidative damage and the underlying molecular mechanisms in human hepatoblastoma cell line HepG2 cells. We found that sedanolide activated the antioxidant response element (ARE)-dependent transcription mediated by the nuclear translocation of NRF2. Pathway enrichment analysis of RNA sequencing data revealed that sedanolide upregulated the transcription of antioxidant enzymes involved in the NRF2 pathway and glutathione metabolism. Then, we further investigated whether sedanolide exerts cytoprotective effects against H_2_O_2_-induced cell death. We showed that sedanolide significantly attenuated cytosolic and mitochondrial reactive oxygen species (ROS) generation induced by exposure to H_2_O_2_. Furthermore, we demonstrated that pretreatment with sedanolide conferred a significant cytoprotective effect against H_2_O_2_-induced cell death probably due to preventing the decrease in the mitochondrial membrane potential and the increase in caspase-3/7 activity. Our study demonstrated that sedanolide enhanced cellular resistance to oxidative damage via the activation of the Kelch-like ECH-associated protein 1 (KEAP1)–NRF2 pathway.

## 1. Introduction

Oxidative stress is defined as an imbalance between oxidants and antioxidants and is associated with the development of various disorders and diseases [1]. Studies have revealed that reactive oxygen species (ROS) generated endogenously or exogenously triggers oxidative stress [2]. Under homeostatic conditions, ROS plays an important role in such physiological cellular processes as proliferation, differentiation, and growth through signaling [3]. In contrast, excess cellular levels of ROS can cause damage to membranes and macromolecules such as proteins, nucleic acids, and lipids, which is closely related to numerous pathological conditions including cancer, inflammatory diseases, and neurodegenerative diseases [4,5,6]. Thus, it is widely believed that the suppression of excessive ROS generation and the resulting oxidative stress is imperative to prevent various diseases.

One of the major antioxidant defense systems is the Kelch-like ECH-associated protein 1 (KEAP1)–nuclear factor E2-related factor 2 (NRF2) signaling pathway [7]. Under physiological conditions, NRF2 is largely bound to KEAP1 in the cytoplasm and constantly degraded through the ubiquitin–proteasome pathway [8]. In the presence of electrophiles or oxidants, NRF2 is released from KEAP1 and translocated into the nucleus where it subsequently binds to antioxidant response element (ARE), thereby initiating the expression of phase II detoxifying and antioxidant enzymes such as NAD(P)H quinone oxidoreductase 1 (NQO1), glutathione *S*-transferase (GST), and superoxide dismutase [7,9,10]. These antioxidant enzymes reduce oxidative stress and consequently attenuate cellular damage. Therefore, the KEAP1–NRF2 pathway plays a pivotal role in the maintenance of cellular function and redox balance. Some botanically derived and synthetic compounds have been found to activate the KEAP1–NRF2 pathway including curcumin, sulforaphane, resveratrol, and so on [11,12,13], and they are in different phases of clinical trials for the treatment of chronic diseases such as cancer and type 2 diabetes mellitus [14]. Thus, the KEAP1–NRF2 pathway is a potential therapeutic target for treating disorders and diseases driven by oxidative stress [15].

**Figure 1:**
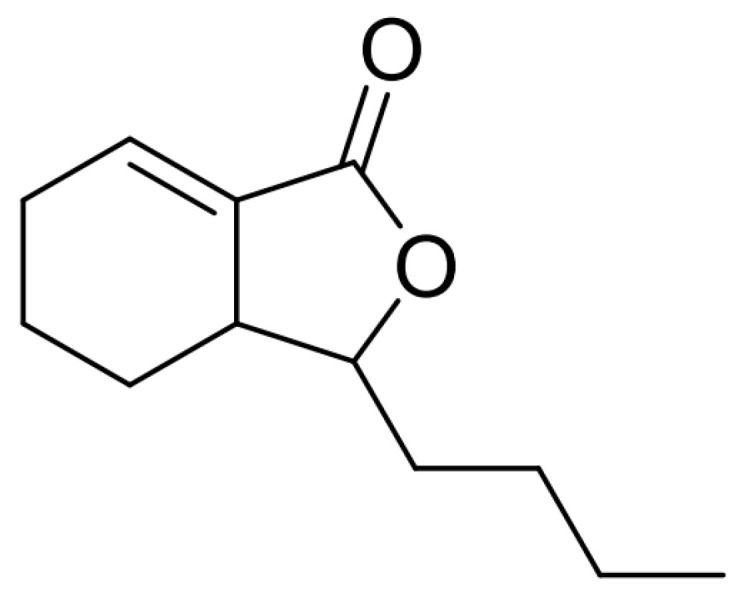
Chemical structure of sedanolide.

Phthalides are a class of bioactive natural products that are widely distributed in plants, fungi, lichens, and liverworts [16]. Recently, these compounds have attracted much attention owing to their complicated chemical structures and various pharmacological activities including antimicrobial, anti-inflammatory, antitumor, and antidiabetic activities [17]. Sedanolide (Figure 1) is a phthalide-like compound and one of the main constituents of the volatile oil of celery [18,19]. It was found to have mosquitocidal, nematicidal, and antifungal activities [20]. In addition, sedanolide exhibits moderate anti-inflammatory effects through the inhibition of cyclooxygenases 1 and 2, which play crucial roles in the inflammatory process [21]. An in vivo study suggested that sedanolide exhibits anticarcinogenic activity in female mice [22]. Most recently, Li et al. reported that sedanolide protects mice against acetaminophen-induced liver injury by activating the Nrf2 pathway [23]. Thus, although this compound may have the potential to prevent disorders and diseases, the precise mechanism by which it modulates intracellular signaling pathways is unknown.

In this study, we show that sedanolide is a KEAP1–NRF2 pathway activator that upregulates the expression of antioxidant genes, using a cell-based assay and RNA sequencing (RNA-seq) analysis. We also demonstrate that sedanolide confers cytoprotective effects against apoptotic cell death and prevents the decrease in the mitochondrial membrane potential (MMP) and the increase in caspase-3/7 activity induced by hydrogen peroxide (H_2_O_2_)-mediated oxidative stress.

## 2. Results

### 2.1. Sedanolide Activates ARE-Dependent Transcription

**Figure 2:**
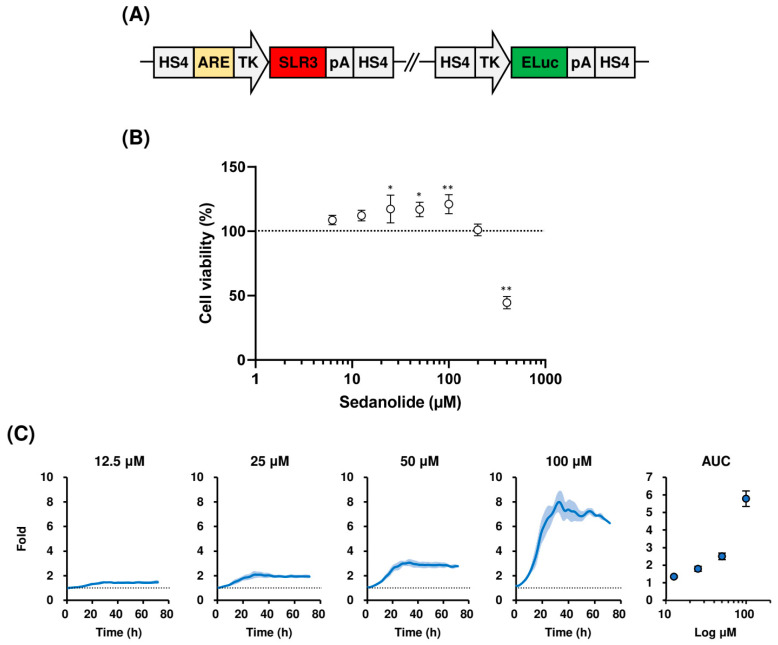
Effects of sedanolide on viability and ARE-driven bioluminescence of luciferases-expressing HepG2 cells. (**A**) Schematic drawing of reporter plasmid. HS4, HS4 insulator; TK, TK promoter; ARE, antioxidant response element; pA, polyA signal. (**B**) Effects of sedanolide on cell viability measured by WST-1 assay. The results are expressed as percent of untreated controls (dotted horizontal line). Values are means ± SD, *n* = 3, one-way ANOVA followed by Tukey’s multiple comparison test. * *p* < 0.05, ** *p* < 0.01, compared with untreated control. (**C**) Real-time bioluminescence recordings of sedanolide-treated luciferases-expressing HepG2 cells. The cells were seeded in a 96-well white clear-bottom plate and treated with sedanolide at various concentrations. Bioluminescence was recorded in real time for 5 s at 30-min intervals for 72 h. The time-dependent change in ARE activation is expressed as a fold change where the relative bioluminescence intensity (SLR3/ELuc) of sedanolide-treated cells was normalized to that of untreated control cells at each time point. Dose dependencies are summarized in the area under the curves (AUCs) at the rightmost panel. Values are means ± SD, *n* = 3.

To determine whether sedanolide modulates the KEAP1–NRF2 pathway, we used the human hepatoblastoma cell line HepG2 in which activation of the KEAP1–NRF2 pathway can be monitored by the ARE-dependent transactivation of the luciferase gene [24]. Red-emitting beetle luciferase (SLR3) was used for monitoring ARE-dependent transcription, and green-emitting beetle luciferase (ELuc) was used as an internal control to normalize SLR3 luminescence and to monitor viability (Figure 2A). We performed measurements in the sedanolide concentration range of 12.5 to 100 μM because sedanolide did not show significant cytotoxicity in luciferases-expressing HepG2 cells measured by the water-soluble tetrazolium salt (WST-1) assay (Figure 2B). Real-time bioluminescence measurement revealed that sedanolide dose-dependently activated ARE-dependent transcription (Figure 2C). The ARE-dependent transcription in 100 μM sedanolide-treated cells was approximately 8-fold higher than that in untreated cells. In addition, whereas SLR3 luminescence intensity was increased following treatment with sedanolide in a dose-dependent manner (Supplementary Figure S1A), ELuc luminescence intensity did not significantly change (Supplementary Figure S1B), indicating that sedanolide does not nonspecifically upregulate overall transcription but specifically activates ARE-dependent transcription without producing toxicity.

### 2.2. Sedanolide Induces Nuclear Translocation of NRF2

**Figure 3:**
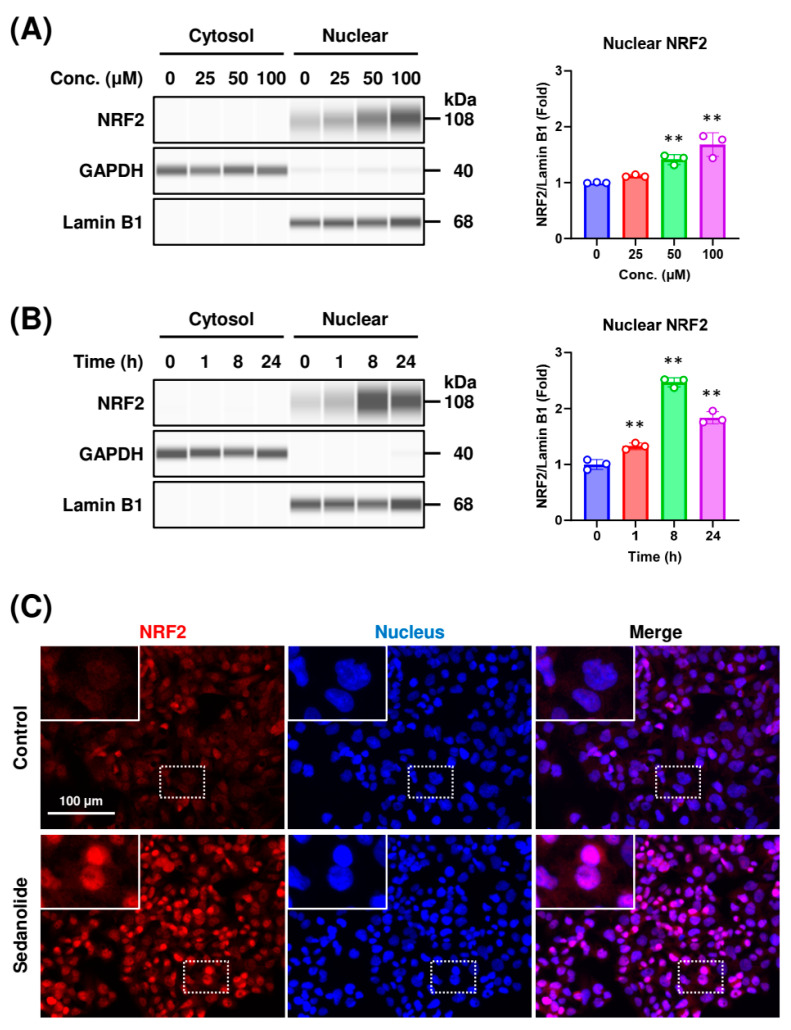
Effects of sedanolide on nuclear localization of NRF2 protein. (**A**) Western blot analysis of cytoplasmic and nuclear NRF2 protein in luciferases-expressing HepG2 cells treated with different concentrations of sedanolide for 24 h. (**B**) Western blot analysis of cytoplasmic and nuclear proteins in cells treated with 100 μM sedanolide for the indicated times. GAPDH and lamin B1 were used as loading control for cytoplasmic and nuclear proteins, respectively. Histogram plots (right panels) show the levels of NRF2 in the nuclei normalized to lamin B1. Values are means ± SD, *n* = 3, one-way ANOVA followed by Tukey’s multiple comparison test. ** *p* < 0.01, compared with untreated control. (**C**) Immunofluorescence analysis of NRF2 proteins. The cells were treated with 100 μM sedanolide for 24 h. Then, the cells were fixed with 4% PFA and permeabilized with 0.1% Triton X-100. NRF2 was probed with a primary anti-NRF2 antibody and visualized with a goat anti-rabbit IgG Alexa Fluor 594 secondary antibody. Nuclei were stained with NucBlue. Fluorescence images were obtained by using a BZ-X710 fluorescence microscope. Insets show high-magnification images of the boxed areas.

The transactivation of ARE-mediated genes requires the translocation of NRF2 into the nucleus [25]. To determine whether the nuclear translocation of NRF2 is induced by the treatment with sedanolide, intracellular localization in sedanolide-treated cells was examined by Western blot analysis (Figure 3A,B) and immunofluorescence staining (Figure 3C). Luciferases-expressing HepG2 cells were exposed to various concentrations of sedanolide for 24 h, and nuclear and cytoplasmic NRF2 levels were determined by treatment with specific antibodies. As shown in Figure 3A, sedanolide dose-dependently increased the nuclear translocation of NRF2. To further verify this result, we examined the dynamics of NRF2 in a time-dependent manner following exposure to sedanolide. Sedanolide induced the nuclear translocation of NRF2 as early as 1 h after treatment, and the translocation reached its peak at 8 h (Figure 3B). In addition, we utilized fluorescence microscopy to further confirm the sedanolide-induced nuclear translocation of NRF2. Upon treatment with sedanolide, the fluorescence signals from NRF2 (red) and nuclei (blue) almost overlapped, demonstrating that NRF2 was translocated into the nucleus by the treatment with sedanolide (Figure 3C). Collectively, these results indicated that sedanolide triggers the nuclear translocation of NRF2.

### 2.3. Sedanolide Activates the NRF2 Pathway

**Figure 4:**
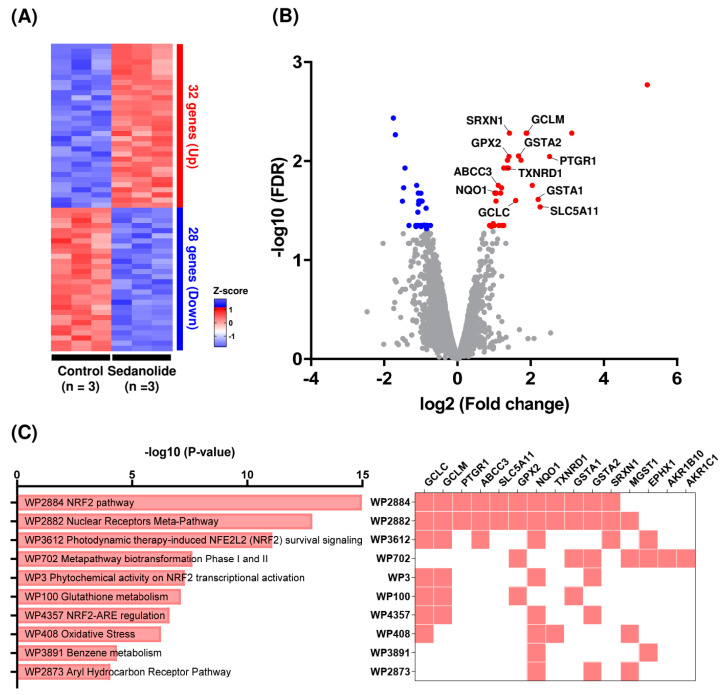
Identification of significant DEGs induced by sedanolide in luciferases-expressing HepG2 cells. (**A**) The number of significant DEGs. Red: upregulation, blue: downregulation. (**B**) Volcano plot of RNA-seq results of sedanolide-treated cells versus control cells. Differential gene expression with log2-normalized fold changes (FCs) in sedanolide-treated cells (*n* = 3) or control cells (*n* = 3) plotted against −log10 FDR. Red dots: upregulated significant DEGs in sedanolide-treated cells (FC > 1.5, FDR < 0.05); blue dots: downregulated significant DEGs in sedanolide-treated cells (FC < −1.5, FDR < 0.05). (**C**) Pathway enrichment analysis in sedanolide-treated cells compared with control cells, as determined by the WikiPathways analysis. Upregulated genes belonging to the corresponding pathways are summarized in the right panel.

To further investigate the biological effects of sedanolide, we analyzed the transcriptomic changes of luciferases-expressing HepG2 cells treated with sedanolide for 24 h and compared them with those of control cells. RNA-seq analysis indicated that 32 genes were upregulated and 28 genes were downregulated (fold change > 1.5) in the sedanolide-treated cells (Figure 4A). The volcano plot of differentially expressed genes (DEGs) revealed that several NRF2 downstream genes, including *GCLC*, *GCLM*, *PTGR1*, *ABCC3*, *SLC5A11*, *GPX2*, *NQO1*, *TXNRD1*, *GSTA1*, *GSTA2,* and *SRXN1*, were significantly upregulated by the treatment with sedanolide (Figure 4B). In addition, we performed pathway enrichment analysis of DEGs in the sedanolide-treated cells using Wikipathways database [26]. As shown in Figure 4C, critical pathways involved in the antioxidant activity, such as the NRF2 pathway (WP2884) and glutathione (GSH) metabolism (WP100), were upregulated by the treatment with sedanolide, suggesting that sedanolide treatment induces antioxidant properties in the cells.

### 2.4. Sedanolide Protects against H_2_O_2_-Induced Cell Death

**Figure 5:**
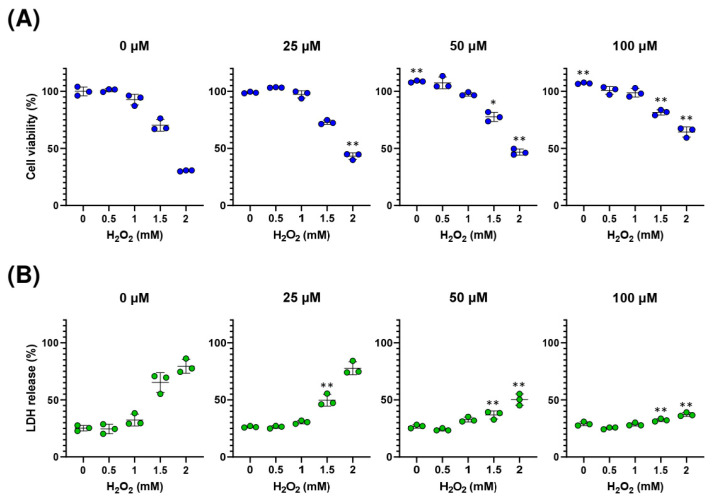
Protective effect of sedanolide on H_2_O_2_-induced cell death in luciferases-expressing HepG2 cells. (**A**) WST-1 assay of H_2_O_2_-induced cytotoxicity in cells pretreated or not pretreated with sedanolide. (**B**) LDH release assay of H_2_O_2_-induced cell membrane damage in cells pretreated or not pretreated with sedanolide. The cells were pretreated with sedanolide at various concentrations for 24 h and then exposed to H_2_O_2_ for an additional 24 h. Values are means ± SD, *n* = 3, two-way ANOVA followed by Tukey’s multiple comparison test. * *p* < 0.05, ** *p* < 0.01, compared with cells exposed to H_2_O_2_ at the corresponding concentrations without sedanolide pretreatment.

As the above-mentioned results led us to speculate that sedanolide could confer tolerance to oxidative stress, we determined whether sedanolide exerts a cytoprotective effect against H_2_O_2_-induced cell death. Luciferases-expressing HepG2 cells were pretreated with various concentrations of sedanolide for 24 h and then exposed to H_2_O_2_ for an additional 24 h. The H_2_O_2_-induced cell death was assessed in terms of cell viability and cell membrane damage by the WST-1 assay and the lactate dehydrogenase (LDH) release assay, respectively. As shown in Figure 5A, when the cells alone were exposed to H_2_O_2_, their viability significantly decreased in a dose-dependent manner. On the other hand, pretreatment of the cells with sedanolide conferred significant cytoprotective effects against cell death induced by the subsequent treatment with H_2_O_2_. The cytoprotective effects of sedanolide against H_2_O_2_-induced cell death were also assessed by the LDH release assay (Figure 5B). LDH release from cells treated with H_2_O_2_ was significantly decreased in the presence of sedanolide. These results suggested that sedanolide confers significant cytoprotective effects against H_2_O_2_-induced oxidative stress.

### 2.5. Sedanolide Reduces Cytosolic and Mitochondrial ROS Levels Increased by H_2_O_2_

**Figure 6:**
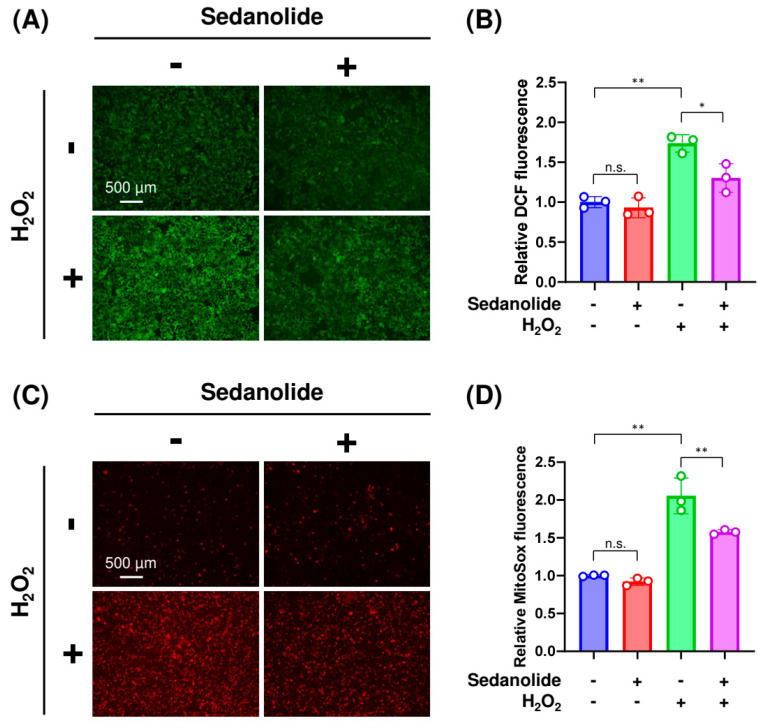
Effects of sedanolide on cytosolic and mitochondrial ROS production in luciferases-expressing HepG2 cells. (**A**,**B**) Effect of sedanolide on cytosolic ROS production in H_2_O_2_-treated cells. The cells were pretreated with sedanolide for 24 h and then exposed to 2 mM H_2_O_2_ for an additional 4 h. Then, the cells were stained with 2’,7’-dichlorodihydrofluorescein diacetate (DCFH-DA) and visualized under a fluorescence microscope (**A**) or analyzed by flow cytometry (**B**). (**C**,**D**) Effect of sedanolide on mitochondrial ROS production in H_2_O_2_-treated cells. The cells were pretreated with sedanolide for 24 h and then exposed to 2 mM H_2_O_2_ for an additional 24 h. Then, the cells were stained with MitoSOX Red and visualized under a fluorescence microscope (**A**) or analyzed by flow cytometry (**B**). Values are means ± SD, *n* = 3, one-way ANOVA followed by Tukey’s multiple comparison test. * *p* < 0.05, ** *p* < 0.01. n.s., not significant.

Based on the above results (Figure 5A,B left panels), a concentration of 2 mM H_2_O_2_ was selected for all subsequent experiments to induce H_2_O_2_-mediated oxidative stress. To investigate whether sedanolide reduces oxidative stress, luciferases-expressing HepG2 cells were pretreated with sedanolide for 24 h and then exposed to 2 mM H_2_O_2_ to detect cytosolic or mitochondrial ROS levels. As shown in Figure 6A, whereas a bright signal of 2′7′-dichlorofluorescein (DCF), which shows enhanced fluorescence during ROS generation, was observed in the H_2_O_2_-exposed cells (left bottom panel), the signal intensity was decreased by the pretreatment with sedanolide (right bottom panel). To quantify the effect of sedanolide on the cytosolic ROS levels, the fluorescence intensities of DCF were measured by flow cytometry (Figure 6B). Consistent with the observation by fluorescence microscopy, H_2_O_2_-induced ROS production was statistically attenuated by the pretreatment with sedanolide. Furthermore, mitochondrial ROS levels were assessed by using MitoSOX Red as the fluorescence probe and analyzed by fluorescence microscopy and flow cytometry. Consistent with the results of the cytosolic ROS levels, pretreatment with sedanolide decreased the production of mitochondrial ROS, which is increased in H_2_O_2_-treated cells (Figure 6C,D). We noted that the 24 h treatment with sedanolide did not cause any appreciable variation in the basal levels of both cytosolic and mitochondrial ROS. These results suggested that the cellular antioxidant capacity is enhanced by the treatment with sedanolide, probably owing to the upregulation of the NRF2 pathway and glutathione metabolism, as shown in Figure 4.

### 2.6. Sedanolide Alleviates H_2_O_2_-Induced Apoptosis

**Figure 7:**
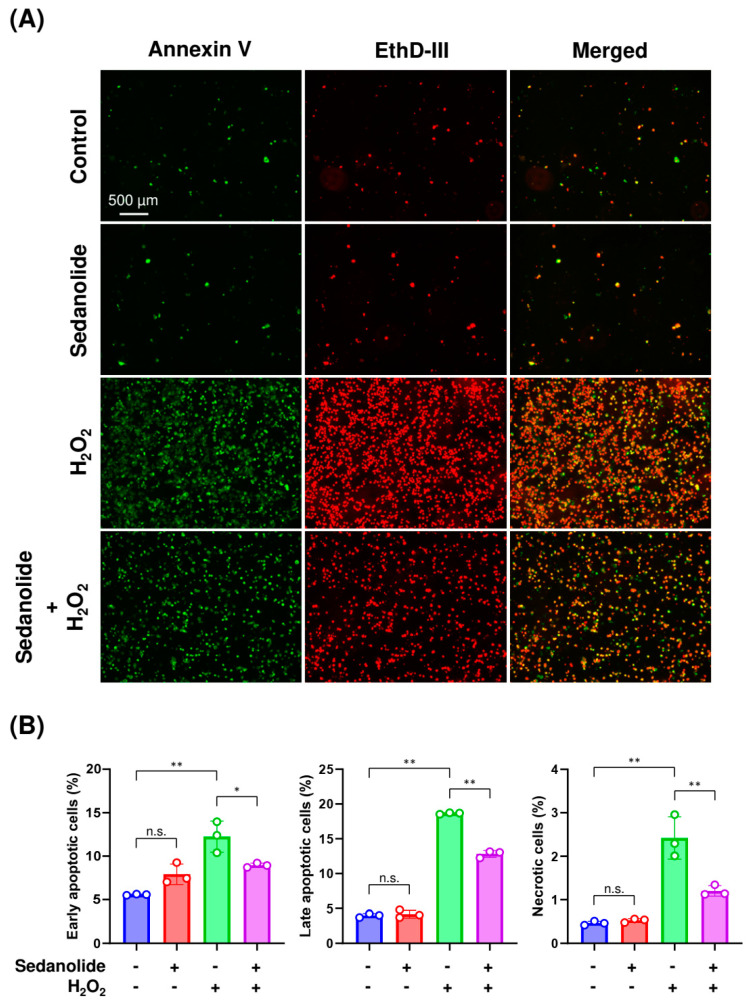
Inhibitory effect of sedanolide on H_2_O_2_-induced apoptosis in luciferases-expressing HepG2 cells. (**A**) Representative images of apoptotic and necrotic cell death by fluorescence microscopy using FITC Annexin V and EthD-III. (**B**) The percentages of early apoptotic, late apoptotic, and necrotic cells were analyzed by flow cytometry. The cells were pretreated with sedanolide for 24 h and then exposed to 2 mM H_2_O_2_ for an additional 24 h. Then, the cells were stained with FITC Annexin V and EthD-III. Values are means ± SD, *n* = 3, one-way ANOVA followed by Tukey’s multiple comparison test. * *p* < 0.05, ** *p* < 0.01. n.s., not significant.

Elevated ROS levels cause extensive and irreparable damage to proteins, nucleic acids, lipids, and organelles, which can lead to activation of the cell death process including apoptosis [27]. We next investigated the cytoprotective effects of sedanolide against H_2_O_2_-induced apoptosis. Luciferases-expressing HepG2 cells were pretreated with sedanolide for 24 h and then exposed to 2 mM H_2_O_2_ for an additional 24 h. Then, the cells were stained with fluorescein isothiocyanate (FITC) Annexin V and Ethidium Homodimer III (EthD-III) and visualized under a fluorescence microscope. As shown in Figure 7A, FITC Annexin V and EthD-III signals could barely be detected in control and sedanolide-pretreated cells. Whereas strong fluorescence signals were visible in response to H_2_O_2_ treatment, these signals were decreased by the pretreatment with sedanolide. We next used flow cytometry to assess the degree of H_2_O_2_-induced apoptosis in the absence or presence of sedanolide pretreatment. When the cells alone were exposed to H_2_O_2_, the numbers of early and late apoptotic cells and necrotic cells were increased, which were significantly decreased by the pretreatment with sedanolide (Figure 7B).

**Figure 8:**
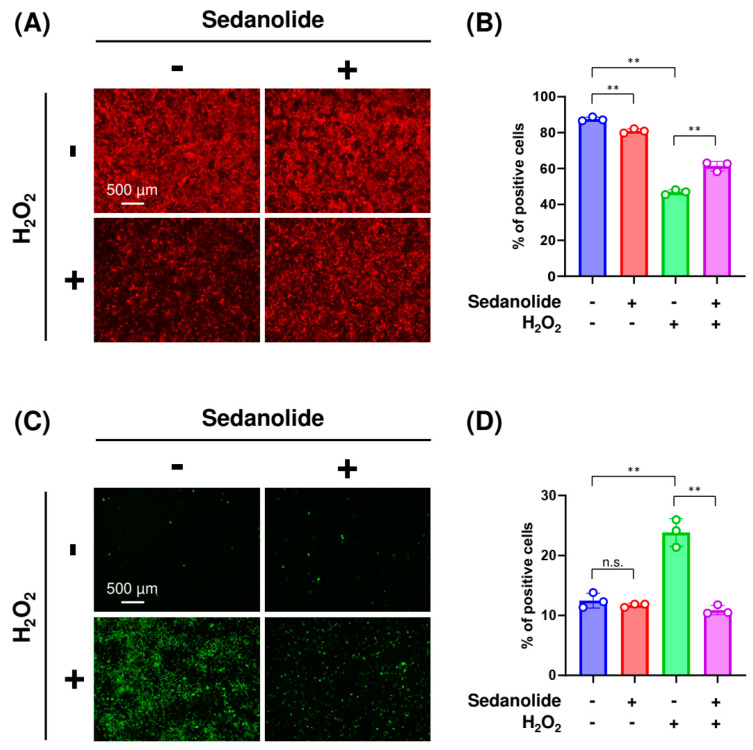
Effect of sedanolide on MMP and caspase-3/7 activity in luciferases-expressing HepG2 cells. (**A**,**B**) Effect of sedanolide on MMP in H_2_O_2_-treated cells. The cells were pretreated with sedanolide for 24 h and then exposed to 2 mM H_2_O_2_ for an additional 24 h. Then, the cells were stained with TMRM and visualized under a fluorescence microscope (**A**) or analyzed by flow cytometry (**B**). (**C**,**D**) Effect of sedanolide on caspase-3/7 activity in H_2_O_2_-treated cells. The cells were pretreated with sedanolide for 24 h and then exposed to 2 mM H_2_O_2_ for an additional 24 h. Then, the cells were stained with CellEvent Caspase-3/7 Green Detection Reagent and visualized under a fluorescence microscope (**C**) or analyzed by flow cytometry (**D**). Values are means ± SD, *n* = 3, one-way ANOVA followed by Tukey’s multiple comparison test. ** *p* < 0.01. n.s., not significant.

The signaling pathways of apoptosis are complex but always rely on the enhanced permeability of mitochondrial membranes and the activation of caspases [28]. To explore the precise mechanisms involved in the anti-apoptotic effect of sedanolide, we further investigated the integrity of MMP and the activity of caspase-3/7 in the H_2_O_2_-treated luciferases-expressing HepG2 cells. MMP status was evaluated using a specific fluorescence probe, tetramethylrhodamine methyl ester (TMRM), which tends to accumulate in healthy mitochondria and produces a red–orange fluorescence signal upon excitation. Fluorescence microscopy observations revealed strong fluorescence in control cells (Figure 8A, left upper panel). Whereas the fluorescence signals were decreased in the H_2_O_2_-treated cells (Figure 8A left bottom panel) owing to the depolarization of the mitochondrial membrane, these signals were retained by the pretreatment with sedanolide (Figure 8A, right bottom panel). Flow cytometric analysis revealed that the number of cells containing healthy mitochondria was decreased by the treatment with 2 mM H_2_O_2_ (Figure 8B). On the other hand, the decrease in the number of healthy mitochondria-containing cells was repressed by the pretreatment with sedanolide.

Caspase-3/7 activity was examined by the CellEvent Caspase-3/7 Green Detection Reagent, which is a nucleic acid-binding dye that harbors the caspase-3/7 cleavage sequence and is fluorescent after being cleaved and bound to DNA. Fluorescence microscopy images showed a significant increase in the intensity of activated caspase-3/7 signals in the H_2_O_2_-treated cells (Figure 8C, left bottom panel) but not in the sedanolide-pretreated cells even in the presence of H_2_O_2_ (Figure 8C, right bottom panel). Flow cytometry was used to quantify the ratio of cells possessing activated caspase-3/7. As shown in Figure 8D, whereas treatment with H_2_O_2_ significantly increased the ratio of cells containing activated caspase-3/7, pretreatment with sedanolide repressed the activation of these caspases. Taken together, these results suggested that sedanolide can protect against H_2_O_2_-induced apoptosis by improving ROS-mediated mitochondrial dysfunction and suppressing the ROS-activated caspase-3/7 signaling pathway.

## 3. Discussion

Phthalides are widely distributed in plants and fungi and exhibit a broad spectrum of biological activities, including antimicrobial, anti-inflammatory, antitumor and antidiabetic activities. However, there are few studies on the bioactivity of sedanolide. In this study, we investigated the cytoprotective effects of sedanolide against H_2_O_2_-induced oxidative stress and explored the mechanism underlying the cytoprotective effects of sedanolide. We found that sedanolide activates the KEAP1–NRF2 pathway, which is followed by the significant upregulation of antioxidant genes. Furthermore, we showed that although H_2_O_2_ treatment decreased cell viability as a result of ROS generation, the pretreatment with sedanolide attenuated the H_2_O_2_-induced mitochondrial damage and apoptotic cell death by reducing cytosolic and mitochondrial ROS generation.

Initially, we revealed that the treatment with sedanolide triggered the nuclear translocation of NRF2 and then activated the ARE-dependent transcription. As sedanolide has an electrophilic α, β-unsaturated carbonyl moiety (Figure 1), it may bind covalently with the thiol group of KEAP1. In general, oxidative stress or electrophilic compounds rapidly lead to the nuclear translocation of NRF2 and the subsequent induction of cytoprotective antioxidant response [29]. After redox homeostasis was achieved, NRF2 was exported from the nucleus to terminate the antioxidant response and then subjected to degradation. Consistent with these observations, we previously found that 5-hydroxy-4-phenyl-butenolide, an NRF2 activator, rapidly increased the ARE-dependent transcription and then gradually decreased it after prolonged incubation [30]. In contrast, as shown in Figure 2C, real-time bioluminescence measurement revealed that the ARE-dependent transcriptional activity increased by the treatment with sedanolide lasted for at least 72 h. Although we do not know the mechanism behind this, we offer a plausible explanation in relation to the nuclear export system. It has been reported that the nuclear export and degradation of NRF2 is controlled by two independent mechanisms involving KEAP1- and Fyn-mediated systems. In the first mechanism, after the induction of NRF2-dependent genes, KEAP1 translocates into the nucleus to mediate the dissociation of NRF2 from ARE and the subsequent formation of the KEAP1–NRF2 complex. This process triggers the export of NRF2 from the nucleus, resulting in the degradation of NRF2 and the termination of NRF2 signaling [31]. In the second mechanism, Fyn, a tyrosine kinase, phosphorylates NRF2 tyrosine 568, which results in the export of NRF2 into the cytosol where NRF2 is degraded by ubiquitination [32]. Hence, it may be possible that sedanolide also inhibits the nuclear translocation of KEAP1 or the phosphorylation of NRF2, leading to the nuclear translocation of NRF2 and the maintenance of ARE-dependent transcriptional activity.

As shown in Figure 4, the mRNA expression levels of antioxidant enzymes belonging to the GSH system, such as *GPX2*, *GCLC*, *GCLM*, *GSTA1*, *GSTA2*, and *MGST1*, were significantly increased by the treatment with sedanolide. The GSH system, a major thiol-dependent antioxidant system, participates in the defense against oxidative stress through the efficient removal of various ROS by glutathione peroxidase (GPX) [33]. Glutamate cysteine ligase (GCL) is the key rate-limiting enzyme in the biogenesis of GSH and is composed of glutamate cysteine ligase catalytic (encoded by *GCLC*) and glutamate cysteine ligase modifier (encoded by *GCLM*) subunits [34]. Whereas *Gclc*-knockout mice were embryonic lethal, the conditional knockout resulted in critical GSH depletion and increased oxidative stress [35]. Lim et al. have reported that the increased oxidative stress in *Gclm*-knockout mice is correlated with decreased capacity for GSH synthesis [36]. On the other hand, GST plays an important role in oxidative stress tolerance including mediating the conjugation of GSH with a wide variety of electrophilic toxic compounds, and its overexpression confers tolerance to H_2_O_2_-induced cell death [37,38]. In this regard, the upregulation of GSH system-related genes is considered to be associated with cytoprotection against oxidative stress and toxic compounds [39,40]. Consistent with these observations, the pretreatment with sedanolide ameliorates H_2_O_2_-induced oxidative stress through the inhibition of cytosolic and mitochondrial ROS accumulation. Taken together, our findings suggest that sedanolide confers tolerance to both oxidative and xenobiotic stress via the upregulation of the GSH system in cells.

In addition to the upregulation of GSH system-related genes, sedanolide increased the transcript levels of *NQO1*, *TXNRD1* (encoding thioredoxin reductase 1), *SRXN1* (encoding sulfiredoxin 1), *AKR1B10* (encoding aldo-keto reductase family 1 member B10), and *AKR1C1* (encoding aldo-keto reductase family 1 member C1). NQO1 plays an important role in protecting cells against oxidative stress by scavenging superoxide and catalyzing the reductive metabolism of chemicals [41]. TXNRD1 also plays a role in preventing oxidative stress by reducing the oxidation states of its substrates such as thioredoxin [42]. SRXN1 detoxifies highly reactive peroxides, including H_2_O_2_, and assumes a crucial role in inhibiting cellular damage triggered by oxidative stress [43]. Aldo-keto reductases, such as AKR1B10 and AKR1C1, are a family of enzymes that catalyze the conversion of aldehydes or ketones into their corresponding alcohols, leading to the inhibition of cytotoxic effects [44]. Therefore, antioxidant enzymes regulated via the NRF2 pathway may be collectively involved in the cytoprotective effects of sedanolide.

The present study also showed that sedanolide suppressed H_2_O_2_-mediated cytosolic and mitochondrial ROS production and attenuated oxidative stress-induced apoptotic cell death. Apoptosis, a programmed cell death, is tightly regulated by a variety of factors and can be divided into extrinsic and intrinsic pathways. The former is initiated by death receptors, whereas the latter is associated with mitochondrial dysfunction [45]. In the mitochondrion-mediated intrinsic apoptotic pathway, oxidative stress induces the depolarization of the mitochondrial membrane, resulting in the loss of MMP as the mitochondrial membrane pore opens. Thereafter, the mitochondria release cytochrome c, which activates the cytoplasmic caspase cascade and induces apoptotic cell death [46]. Thus, the loss of MMP is indicative of mitochondrial dysfunction and is an early event in the onset of mitochondrion-mediated intrinsic apoptosis [47]. To evaluate the preventive effect of sedanolide on mitochondrial dysfunction, MMP values were examined by fluorescence microscopy and flow cytometry (Figure 8A,B). We found that whereas the MMP values were significantly decreased in H_2_O_2_-treated cells, these values were restored through the pretreatment with sedanolide.

We also found that sedanolide repressed the increase in caspase-3/7 activity triggered by the treatment with H_2_O_2_. Caspases are intracellular cysteine protease enzymes that play a major role in the apoptotic mechanism, and caspase-3/7 is the final step in both extrinsic and intrinsic apoptotic pathways [48]. Mouse embryonic fibroblasts lacking caspase-3/7 were resistant to both death receptor-initiated apoptosis and mitochondrion-mediated apoptosis [49]. In addition, it has been reported that caspase-3/7 is required for the induction of efficient apoptosis by the activating feedback amplification of upstream apoptotic signals [50]. Therefore, our findings suggest that the anti-apoptotic effects of sedanolide in H_2_O_2_-treated cells may be partially explained by the reduction in caspase-3/7 activity. However, although pretreatment with sedanolide completely abolished caspase-3/7 activation in H_2_O_2_-treated cells (Figure 8C,D), that only partially inhibited H_2_O_2_-induced apoptosis (Figure 7). These results suggest that sedanolide is ineffective in protecting cells from caspase-independent apoptosis such as apoptosis-inducing factor and endonuclease G-mediated pathways.

In conclusion, our results revealed that sedanolide exerts cytoprotective effects against H_2_O_2_-induced cell death. In addition, sedanolide inhibits excessive ROS generation and mitochondrial dysfunction probably owing to its ability to upregulate antioxidant enzymes belonging to the NRF2 pathway and glutathione metabolism. A recent study has suggested that sedanolide activates the NRF2 pathway [23]. Our study describes for the first time the role of the cytoprotective effects of sedanolide in ameliorating H_2_O_2_-induced cell death. Taken together, the results suggest a potential mechanism underlying the cytoprotective effect of sedanolide.

## 4. Materials and Methods

### 4.1. Generation of Luciferases-Expressing HepG2 Cells

Human hepatoblastoma cell line HepG2 was purchased from the RIKEN BioResource Center (Ibaraki, Japan). Luciferases-expressing HepG2 cells were generated as reported previously [24]. Briefly, internal control reporter plasmid pTK-ELuc-PEST-R4-Bsd [51] carrying herpes simplex thymidine kinase (TK) promoter and green-emitting Emerald Luc (ELuc) [52] (Toyobo, Osaka, Japan) from *Pyrearinus termitilluminans* was integrated into HepG2 cells harboring the multi-integrase mouse artificial chromosome (MI-MAC) vector [53,54] (a gift from Dr. M. Oshimura and Dr. Y. Kazuki of Tottori University) by co-transfection with R4 integrase expression plasmid. The selected cells were further co-transfected with a reporter plasmid pARE-TK-SLR3-ϕC31-Neo [30] carrying five tandem repeats of ARE (5′-TCACAGTGACTCAGCAAAATT-3′), TK promoter, and red-emitting Stable Luciferase Red3 (SLR3) from *Phrixotrix hirtus*, and the ϕC31 integrase expression plasmid and the reporter plasmid was integrated into the ϕC31 site of the MI-MAC vector.

### 4.2. Cell Culture and Treatment

Luciferases-expressing HepG2 cells were cultured in Dulbecco’s Modified Eagle Medium (DMEM; Fujifilm Wako Pure Chemical Co., Osaka, Japan) supplemented with 10% fetal bovine serum (FBS; Sigma-Aldrich, St. Louis, MO, USA), 1% Minimum Essential Medium Non-Essential Amino Acids (MEM NEAA; Gibco, Grand Island, NY, USA), and 1 mM sodium pyruvate (Gibco) at 37 °C in a humidified atmosphere with 5% CO_2_. For experiments, cells were seeded in differently sized multi-well plates at a density of 1 × 10^5^ cells/cm^2^ and incubated for 24 h. Subsequently, the cells were pretreated with sedanolide (LKT laboratories, St. Paul, MN, USA) for 24 h and then exposed to H_2_O_2_ (Fujifilm Wako Pure Chemical Co.) for an additional 24 h unless otherwise noted. Sedanolide was dissolved in dimethyl sulfoxide (DMSO) at 100 mM as a stock solution and stored at −30 °C until used. The final concentration of DMSO did not exceed 0.1% throughout the study.

### 4.3. Cytotoxicity Assay

Cell viability was determined using a Premix WST-1 Cell Proliferation Assay System (Takara Bio, Shiga, Japan) according to the manufacturer’s instructions. Cell membrane damage was assessed using a Cytotoxicity LDH Assay Kit-WST (Dojindo Laboratories, Kumamoto, Japan) following the manufacturer’s instructions. 

### 4.4. Real-Time Bioluminescence Measurement

Luciferases-expressing HepG2 cells were seeded in 96-well white clear-bottom plates at 3 × 10^4^ cells/well. After incubation for one day, the medium was replaced with DMEM without phenol red (Gibco) containing 10% FBS (Hyclone; Thermo Fisher Scientific, Inc., Waltham, MA, USA), 1% Glutamax (Gibco), 1% MEM NEAA, 1 mM sodium pyruvate (Fujifilm Wako Pure Chemical Co.), 25 mM HEPES (pH 7.0; Nacalai Tesque, Kyoto, Japan), 300 μM d-luciferin potassium salt (RESEM B.V., Lijnden, The Netherlands), and sedanolide at various concentrations. Bioluminescence was recorded in real time for 5 s at 30-min intervals in the absence (F0 value) or presence (F2 value) of an R62 long-pass filter (HOYA, Tokyo, Japan) at 37 °C in a humidified atmosphere with 5% CO_2_ using a microplate-type luminometer (WSL-1565 Kronos HT; ATTO, Tokyo, Japan). ELuc and SLR3 luminescence intensities were calculated from F0 and F2 values as described previously [55].

### 4.5. Western Blot Analysis

Luciferases-expressing HepG2 cells were seeded in 6-well plates at 9 × 10^5^ cells/well. After incubation for one day, the cells were treated with various concentrations of sedanolide. Cytoplasmic and nuclear fractions were extracted using NE-PER Nuclear and Cytoplasmic Extraction Reagents (Thermo Fisher Scientific, Inc.) according to the manufacturer’s instructions. Protein concentrations were measured using a Pierce BCA Protein Assay Kit (Thermo Fisher Scientific, Inc.) according to the manufacturer’s instructions. Protein levels were analyzed using the capillary electrophoresis western system Wes (ProteinSimple Co., San Jose, CA, USA) following the manufacturer’s instructions. In brief, the protein samples were mixed with the 5× Fluorescent Master Mix (ProteinSimple Co.) and then heated at 95 °C for 5 min. Next, the heated samples, biotinylated protein ladder, primary antibodies, horseradish peroxide-conjugated anti-rabbit secondary antibody, and luminol-peroxide were loaded into the 12–230 kDa Wes Separation Module (ProteinSimple Co.). Primary antibodies Nrf2 (#NBP1-32822; Novus Biologicals, Centennial, CO, USA), lamin B1 (#12586; Cell Signaling Technology, Danvers, MA, USA), and GAPDH (#2118; Cell Signaling Technology) were diluted to 1:50, 1:50, and 1:100, respectively. Protein levels were analyzed using Compass software (version 5.0.1) for Simple Western (ProteinSimple Co.).

### 4.6. Immunofluorescence Analysis

Luciferases-expressing HepG2 cells were seeded on coverslips placed in 24-well plates at 1.8 × 10^5^ cells/well. After incubation for one day, the cells were treated with 100 μM sedanolide for 24 h. The cells were washed with PBS and fixed with 4% paraformaldehyde (Fujifilm Wako Pure Chemical Co.) for 15 min. Then, the cells were washed three times and permeabilized with 0.1% Triton X-100 for 10 min. After permeabilization, the cells were washed three times with PBS and blocked with 1% BSA for 1 h. After that, the cells were incubated with anti-Nrf2 antibody (1:200 dilution). The cells were washed three times with PBS and incubated for 1 h with goat anti-rabbit IgG Alexa 594 (1:1000 dilution; Thermo Fisher Scientific, Inc.). After washing with PBS, the cells were mounted with ProLong Glass Antifade Mountant with NucBlue Stain (Thermo Fisher Scientific, Inc.). Fluorescence images were obtained on a BZ-X710 fluorescence microscope and BZ-X Viewer (version 1.3.0.5) (Keyence, Osaka, Japan).

### 4.7. RNA Sequencing and Data Analysis

Luciferases-expressing HepG2 cells were seeded in 6-well plates at 9 × 10^5^ cells/well. After incubation for one day, the cells were treated with 100 μM sedanolide for 24 h. Total RNA was extracted using an RNeasy Mini Kit (Qiagen, Valencia, CA, USA) according to the manufacturer’s instructions. RNA-seq analysis was outsourced to Genome-Lead Co. Ltd. (Takamatsu, Japan). In brief, mRNA was purified from total RNA samples using a KAPA mRNA Capture Kit (KAPA Biosystems, Potters Bar, UK). Next, a cDNA library was prepared using an MGIEasy RNA Directional Library Prep Set (MGI Tech, Shenzhen, China). The libraries were sequenced on a DNBSEQ-T7RS platform (MGI Tech) using a DNBSeq-T7RS High-Throughput Sequencing Set (MGI Tech), and 150 bp paired-end reads were generated. The quality of the sequence data was evaluated using FastQC. Then, Trimmomatic was used to trim adaptor sequences and remove low-quality reads. The sequences were aligned to the GRCh38 human reference genome using the alignment tool STAR. Reads in transcripts per million (TPM) formats were computed using RNA-seq by Expectation Maximization (RSEM). To identify DEGs and altered pathways, read counts were uploaded and analyzed using the integrated Differential Expression and Pathway (iDEP.96) web-based applications [56]. The DEGs were identified using the DESeq2 method with a false discovery rate (FDR) cutoff of 0.05 and a minimum fold change of 1.5. The analysis of enriched pathways in DEGs was conducted using the Wikipathways database [26] with the results being compiled in the form of histograms. Data are deposited in the NCBI Gene Expression Omnibus (GEO) database under accession number GSE245428.

### 4.8. Measurement of Cytosolic and Mitochondrial ROS Level

Luciferases-expressing HepG2 cells were seeded in 12-well plates at 3.6 × 10^5^ cells/well. After incubation for one day, the cells were pretreated with sedanolide for 24 h and then exposed to H_2_O_2_ for an additional 4 h. 2′,7′-Dichlorodihydrofluorescein diacetate (DCFH-DA; Sigma-Aldrich), which permeates cells and is oxidized by ROS into fluorescent DCF, was used to assess cytosolic ROS levels. After the treatment with H_2_O_2_, the cells were loaded with 20 μM DCFH-DA for 30 min at 37 °C in a humidified atmosphere with 5% CO_2_. Then, the cells were collected and analyzed using a FACSCalibur flow cytometer (Becton, Dickinson and Company, Franklin Lakes, NJ, USA). For the visualization of DCF fluorescence, the cells were observed under a BZ-X710 all-in-one fluorescence microscope and BZ-X Viewer (version 1.3.0.5).

Luciferases-expressing HepG2 cells were seeded in 12-well plates at 3.6 × 10^5^ cells/well. After incubation for one day, the cells were pretreated with sedanolide for 24 h and then exposed to H_2_O_2_ for an additional 24 h. Fluorescent probe MitoSOX Red (Thermo Fisher Scientific, Inc.) was used to measure mitochondrial ROS production. After the treatment with H_2_O_2_, the cells were stained with 5 μM MitoSOX Red for 30 min at 37 °C in a humidified atmosphere with 5% CO_2_. Then, the cells were collected and analyzed using a FACSCalibur flow cytometer. For the visualization of MitoSOX Red, the cells were observed under a BZ-X710 all-in-one fluorescence microscope and BZ-X Viewer (version 1.3.0.5).

### 4.9. Apoptosis Assay

Luciferases-expressing HepG2 cells were seeded in 12-well plates at 3.6 × 10^5^ cells/well. After incubation for one day, the cells were pretreated with sedanolide for 24 h and then exposed to H_2_O_2_ for an additional 24 h. Cell death induced by H_2_O_2_ was assessed using an Apoptotic/Necrotic Cells Detection Kit (Promokine, Heidelberg, Germany) according to the manufacturer’s instructions. In brief, the cells were stained with FITC Annexin V and EthD-III for 15 min in the dark at room temperature. Cellular status was analyzed using a FACSCalibur flow cytometer. Data were collected from at least 10,000 gated events. Cellular status was determined as follows: early apoptosis (Annexin V positive/EthD-III negative), late apoptosis (Annexin V positive/EthD-III positive), necrosis (Annexin V negative/EthD-III positive), and healthy cells (Annexin V negative/EthD-III negative). For the visualization of FITC Annexin V and EthD-III fluorescence, the cells were observed under a BZ-X710 all-in-one fluorescence microscope and BZ-X Viewer (version 1.3.0.5).

### 4.10. Measurement of Mitochondrial Membrane Potential Changes

Luciferases-expressing HepG2 cells were seeded in 12-well plates at 3.6 × 10^5^ cells/well. After incubation for one day, the cells were pretreated with sedanolide for 24 h and then exposed to H_2_O_2_ for an additional 24 h. TMRM (Thermo Fisher Scientific, Inc.), a cationic fluorescent probe taken up by mitochondria in a membrane potential-dependent manner, was used to assess MMP. After the treatment with H_2_O_2_, the cells were loaded with 100 nM TMRM for 30 min at 37 °C in a humidified atmosphere with 5% CO_2_. Then, the cells were collected and analyzed using a FACSCalibur flow cytometer. For the visualization of TMRM, the cells were observed under a BZ-X710 all-in-one fluorescence microscope and BZ-X Viewer (version 1.3.0.5).

### 4.11. Quantification of Caspase-3/7 Activity

Luciferases-expressing HepG2 cells were seeded in 12-well plates at 3.6 × 10^5^ cells/well. After incubation for one day, the cells were pretreated with sedanolide for 24 h and then exposed to H_2_O_2_ for an additional 24 h. The percentage of cells containing active caspase-3/7 was determined using CellEvent Caspase-3/7 Green Detection Reagent (Thermo Fisher Scientific, Inc.) according to the manufacturer’s instructions. After the treatment with H_2_O_2_, the cells were loaded with 5 μM reagent for 30 min at 37 °C in a humidified atmosphere with 5% CO_2_. Then, the cells were collected and analyzed using a FACSCalibur flow cytometer. For the visualization of fluorescence signals, the cells were observed under a BZ-X710 all-in-one fluorescence microscope and BZ-X Viewer (version 1.3.0.5).

### 4.12. Statistical Analysis

All assays were conducted in triplicate at least. Data are expressed as means ± standard deviations (SDs). Comparisons were performed by one-way analysis of variance (ANOVA) or two-way ANOVA followed by Tukey’s multiple comparison test. GraphPad Prism 8.4.3 (GraphPad Software Inc., San Diego, CA, USA) was used for statistical analysis, and a *p* value less than 0.05 was considered statistically significant.

## Data Availability

Data are contained within the article and supplementary materials.

## Supplementary Materials

The following supporting information can be downloaded at: https://www.mdpi.com/article/10.3390/ijms242216532/s1.
