# Supplementary material for: Sedanolide Activates KEAP1–NRF2 Pathway and Ameliorates Hydrogen Peroxide-Induced Apoptotic Cell Death
Source: Int J Mol Sci. 2023 Nov 20;24(22):16532. doi: 10.3390/ijms242216532 (PMC10671709; doi:10.3390/ijms242216532)
Supplement: Supplementary file 1 [file ijms-24-16532-s001.zip › ijms-2697936-supplementary.pdf]

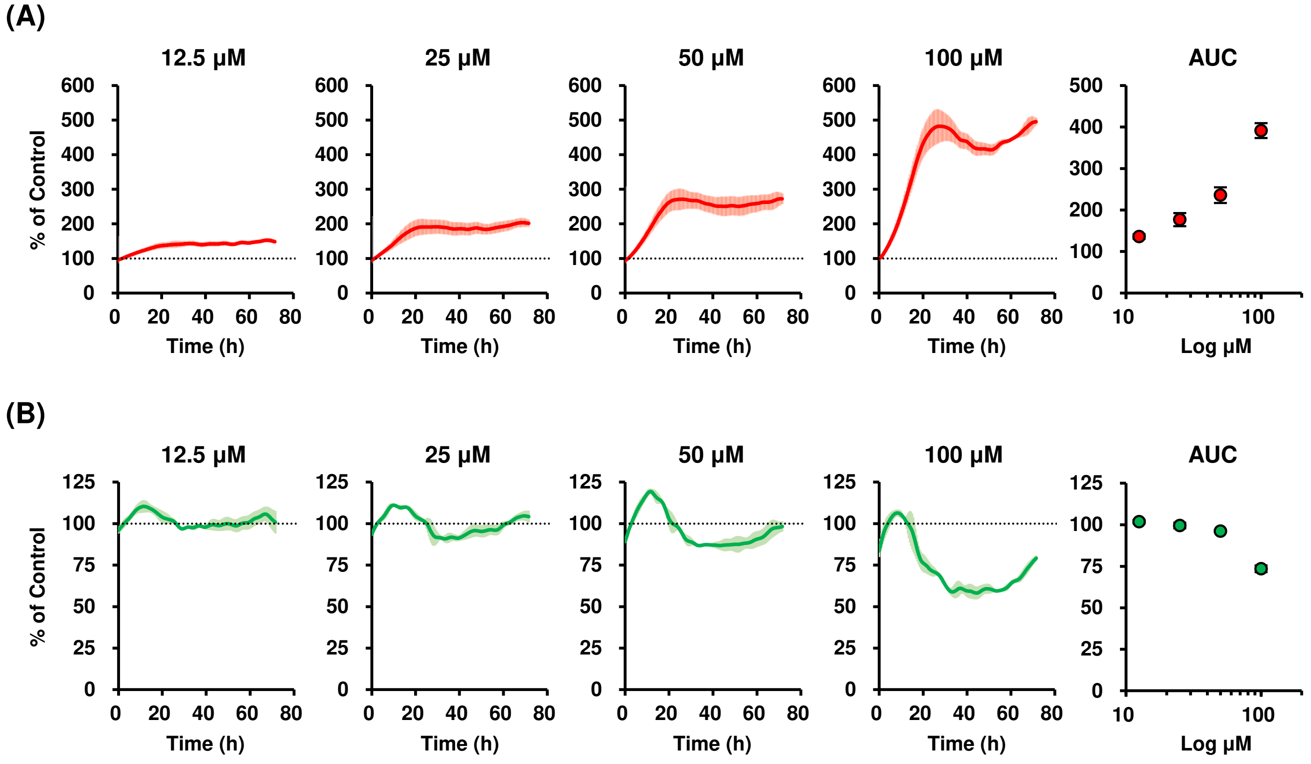

Supplementary Figure S1. Real-time bioluminescence recording of sedanolide-treated luciferases-expressing HepG2 cells. Red and green plots represent ARE-TK-driven and TK-driven light outputs of (A) SLR3 and (B) ELuc, respectively. The cells were seeded in a 96-well white clear-bottom plate and treated with sedanolide at various concentrations. Bioluminescence was recorded in real time for 5 s at 30-min intervals for 72 h. Values are means ± SD, n = 3.
